# Fluorapatite-Coated Percutaneous Devices Promote Wound Healing and Limit Epithelial Downgrowth at the Skin-Device Interface

**DOI:** 10.1155/2023/2212035

**Published:** 2023-03-29

**Authors:** Samantha K. Steyl, James Peter Beck, Jayant P. Agarwal, Kent N. Bachus, David L. Rou, Sujee Jeyapalina

**Affiliations:** ^1^Orthopaedic and Plastic Surgery Research Laboratory, George E. Wahlen Department of Veterans Affairs Medical Center, Salt Lake City, Utah 84148, USA; ^2^Division of Plastic Surgery, Department of Surgery, University of Utah School of Medicine, Salt Lake City, Utah 84112, USA; ^3^Department of Biomedical Engineering, University of Utah, Salt Lake City, Utah 84112, USA; ^4^Department of Orthopaedics, University of Utah School of Medicine, Salt Lake City, Utah 84112, USA

## Abstract

A percutaneous osseointegrated device becomes deeply ingrown by endosteal bone and traverses the overlying soft tissues of the residual limb, providing a direct link to the bone-anchored artificial limb. Continuous wound healing around these devices can result in the formation of sinus tracts as “down-growing” epithelial cells are unable to recognize and adhere to the “nonbiological” implant surface. Such sinus tracts provide paths for bacterial colonization and deep infection. In order to limit adverse outcomes and provide a robust seal, it was hypothesized that by coating the titanium surface of the percutaneous post with the mineral component of dental enamel, down-growing epidermal cells might recognize the coating as “biological” and adhere to this nonliving surface. To test this hypothesis, sintered partially and fully fluoridated hydroxyapatite (HA) was chosen as coatings. Using an established surgical protocol, fluorapatite (FA), hydroxyfluorapatite (FHA), HA-coated percutaneous posts, and titanium controls were surgically placed under the dorsal skin in 20 CD hairless rats. The animals were sacrificed at four weeks, and implants and surrounding tissues were harvested and subjected to further analyses. Downgrowth and granulation tissue area data showed statistically significant reductions around the FA-coated devices. Moreover, compared to the control group, the FA- and HA-coated groups showed downregulation of mRNA for EGFr, EGF, and FGF-10. Interestingly, the FA-coated group had upregulation of TGF-*α*. These data suggest that FA could become an ideal coating material for preventing downgrowth, assuming the long-term stability of these coated surfaces can be verified in a clinically relevant animal model.

## 1. Introduction

Percutaneous osseointegrated devices are currently used clinically for the skeletal docking of prosthetic limbs. Because these devices traverse the protective skin barrier, infection remains a leading cause of device failure. It is presumed that the lack of integration (or attachment) of the surrounding epithelial tissue with the device surface results in a continuous ongoing wound healing process [1]. Subsequently, as a result of this altered wound healing, sinus tracts form around the percutaneous implant in which opportunistic pathogens can persist, evading cellular immune responses and personal hygiene measures. Although attendant superficial infections can be treated with either topical, oral, or systemic antimicrobial agents, frequent and prolonged antibiotic therapy can select for more virulent bacteria that are antibiotic-resistant [2, 3]. Thus, over longer implant in situ times, the tissues that surround these percutaneous devices can become infected with antibiotic-resistant bacterial strains resulting in osteomyelitis and subsequent device removal. In one clinical series, up to 55% infection rates have been reported [4]. It is believed that the success of percutaneous devices could be improved by selecting appropriate surfaces or device designs that allow/promote the integration of the surrounding tissue with the surface of the implant, thus creating a seal against deep bacterial invasion.

When trying to devise a material system that met the requirement for surface attachment, natural percutaneous interfaces such as tusks, teeth, and fingernails were analyzed. It is interesting to note that these organs are made up of either keratin or apatite [5–8]. Most importantly, apatite-based surfaces, such as those in tooth enamel and tusks, remain permanently integrated with the surrounding tissues. These surfaces are fluoridated forms of hydroxyapatite (HA; Ca10(PO4)6(OH)2)—fluorapatite (FA; Ca10(PO4)6F2) or fluorohydroxyapatite (FHA; Ca10(PO4)6 Fy(OH)2-y). To emphasize, while human tooth enamel is comprised of a carbonated form of FHA arranged in rods and with a low organic material content [9, 10], shark tooth enamel is composed of FA. It has been reported that the substitution of hydroxide groups with fluoride groups increases the mechanical strength, stiffness, and bulk modulus of the apatites [9]. It has also been reported that at the gingival-enamel interface, so-called junctional epithelial gingival cells form a direct connection to the enamel via hemidesmosomes [11, 12]. This interface is further stabilized by Sharpey's fibers. Many researchers have indicated that junctional epidermal cells are induced by the enamel surfaces (FA/FHA surface) to express hemidesmosomes that subsequently allow them to adhere to the nonliving enamel surface [13]. This knowledge led us to investigate the fluoridated apatites as coating substrates for percutaneous device applications.

Our previous in vitro study was initially designed to characterize the in-house synthesized apatite powders and analyze the adhesion and differentiation properties of keratinocytes and fibroblasts on these surfaces when compressed and sintered [14]. The literature indicates that FA reaches a liquid phase at 1080°C [15] and undergoes a phase transformation at higher temperatures (between 1300°C and 1400°C) to tricalcium phosphates [14, 16, 17]. Our previous study showed a significant increase in adhesion and differentiation of keratinocytes on FA surfaces sintered at 1150°C compared to Ti and HA surfaces. Although FA sintered at 1150°C was able to promote differentiation of keratinocytes under in vitro conditions, the question remained whether this finding would occur under in vivo conditions.

The purpose of this study was, therefore, to investigate the in vivo efficacy of sintered FA coatings when used for percutaneous device applications to allow for the completion of soft tissue wound healing at the skin-device interface (i.e., stoma). Based on the in vitro data [14], it was hypothesized that sintered FA and FHA surfaces would improve wound healing outcomes (i.e., reduced downgrowth and granulation tissue formation) at the interface when compared to the current standard of care percutaneous device materials, titanium, and HA-coated titanium. This hypothesis was tested in an established rat-back model. It is believed that if a stable biological seal at the stoma can be maintained with these coated surfaces, then the infection risk and extended wound healing processes around these percutaneous devices could be drastically reduced, resulting in a healed stoma with limited down growth and mature granulation tissue at the interface.

## 2. Methods and Materials

### 2.1. Device Fabrication

Percutaneous devices were designed at the University of Utah and manufactured at Thortex, Inc. (Portland, OR). The devices consisted of a circular subdermal base (∼17 mm in diameter) that sits beneath the hypodermis and anchors the implant to the body via a porous coating that facilitates fibrous soft tissue interdigitation. A cylindrical percutaneous post (5 mm in diameter and 10 mm in height) protrudes through the skin. The subdermal fixation surfaces and deeper 5 mm portions of the percutaneous posts of each device were coated with 1 mm thick, commercially pure titanium (Ti) porous coating (P2 coating type; Thortex, Portland, OR). Finally, all devices were cleaned, passivated, and sterilized prior to further coating and surgical implantation using ASTM standard B600-91.

### 2.2. Device Coatings

The apatite (HA, FHA, and FA) powders were manufactured in-house, in accordance with the methods previously described [18, 19]. After synthesis, the powders were characterized using x-ray diffraction (XRD), inductively coupled plasma-mass spectroscopy (ICP-MS), and Fourier-transform infrared spectroscopy (FTIR) to confirm the apatite structure [14]. The fluoride contents were calculated using a commercially available fluoride probe (E41M017; Radiometer Analytical, Hach, Loveland, CO) [14]. After characterization, all powders were first sintered at 1150°C and were manually sieved to obtain coating granules with grain sizes between 60-250 *μ*m. Sterilized devices and powders were then sent to N2 Biomedical (Bedford, MA) for coating using their proprietary IonTite process. In order to remove nonadherent particles, after the devices were coated with apatite, they were placed in 70% ethanol on a rocking table for 15 minutes and then allowed to dry. The devices were then prepared for surgery following a low-temperature ethylene oxide gas clinical sterilization process.

### 2.3. Surgical Procedure

A total of twenty, two-month-old female CD hairless rats (strain 184; Charles River Laboratories, Wilmington, MA) were used for the study. A local institutionally approved IACUC protocol (SLC VA IACUC #A1701) was used as the guidance document for conducting the surgeries and subsequent animal care. Briefly, twenty rats were assigned to one of four groups based on the implant type: Group 1 (uncoated titanium control; *n* = 5), Group 2 (HA-coated; *n* = 5), Group 3 (FHA-coated; *n* = 5), and Group 4 (FA-coated; *n* = 5). The rats were prepared for surgery by scrubbing the back three times with alternating passages of ethanol and Betadine scrub and finally with Betadine solution. The midline of the back and scapulae were marked with a surgical marking pen. A longitudinal incision was made parallel to the midline, and a single subdermal pocket was created under the skin with blunt-tipped scissors. A stoma was then made in the area above the pocket with a biopsy punch to facilitate the fitting of the percutaneous post. Next, a single device (uncoated titanium control, HA-coated, FHA-coated, or FA-coated) was placed in the pocket, and the post was fitted through the stoma. The incision was then closed with sutures, and the wounds (i.e., stoma and incision line) were protected with nonadherent absorbent pads (Telfa; Covidien, Mansfield, MA) and covered with breathable TegadermTM transparent film dressings (3M, Maplewood, MN). Rats were individually housed in standard shoebox cages and freely ambulated for 28 days after surgery, at which point they were sacrificed. Food and water were provided ad libitum with a standard 12 h:12 h light:dark cycle. At necropsy, the implant and surrounding tissues were harvested and processed for tissue histology or polymerase chain reaction (PCR), or immunofluorescence (IF) staining.

### 2.4. PCR Assays

**Figure 1:**
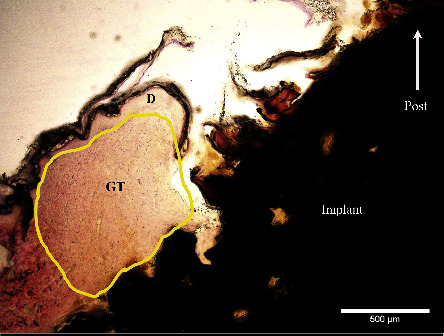
A representative H&E-stainedskin-implant interface cross-section showing granulation tissue (GT), dermis (D), implant, and post.

At necropsy, the granulation tissue from the periprosthetic region was isolated (Figure 1, granulation tissue), infiltrated with RNAlater (Invitrogen, Carlsbad, CA), and stored at −80°C. The total RNA was extracted from frozen granulation tissues using the RNeasy® Fibrous Tissue Mini Kit (Qiagen GmbH, Hilden, Germany). The quality and quantity of RNA were determined using a spectrophotometer (Hitachi, U-2000 Spectrophotometer, Japan) and were reverse transcribed with an RT2 First Strand Kit (Qiagen GmbH, Hilden, Germany). The resulting cDNA was diluted with RNase-free ultrapure water and stored at −20°C and was subjected to quantitative PCR studies to confirm the upregulation and downregulation of epidermal growth factor (EGF), epidermal growth factor receptor (EGFr), fibroblast growth factor 10 (FGF-10), and transforming growth factor *α* (TGF-*α*) genes using real-time RT-PCR. The forward and backward primers for EGFr (Cat. no: PPR48960 A), EGF (Cat. no: PPR43509 B), FGF-10 (Cat no. PPR06632 A), and TGF-*α* (PPR06486 A) were purchased from Qiagen GmbH, Hilden, Germany. Real-time RT-PCR was performed in triplicates using 18 *μ*l of Power SYBR Green PCR Master Mix (Thermo Fisher Scientific, Carlsbad, CA), forward and reverse primers mixture, and 2 *μ*l of cDNA by following the manufacturers' recommended procedure. The Ct values for all samples were normalized to GAPDH expression level of each respective plate. An average value of three runs/groups was used for statistical analysis. A RNA sample from a single titanium control animal was used as the internal control in each PCR plate run to ensure repeatability between runs and to validate accuracies/errors between the PCR runs. Fold-change was calculated using 2^−ΔΔ^Ct values. The level of mRNA expressions for all four genes for the FA group was compared to the expression levels for both the Ti implants and the HA implants.

### 2.5. Confocal Analyses

Fresh-frozen tissue samples were sectioned on a cryostat machine at a thickness of 12 *μ*m, transferred to charged microscope slides, and frozen at −80°C until further analysis. For staining, slides were allowed to air dry for 10 minutes at room temperature (RT). Sections were permeabilized with a solution of 0.3% Triton X-100 in TBS for 10 minutes. They were then blocked with a blocking solution (BlockAid Blocking Solution, Thermo Fisher Scientific, Waltham, MA, USA) for 1 h at room temperature. Next, sections were incubated overnight at 4°C with the primary antibodies as follows: anti-TGF*α* (Prestige Antibodies, Sigma-Aldrich, St. Louis, MO, USA) and anti-EGFr (abcam, Cambridge, UK). The following day, samples were washed 3 × 5 minutes in TBS, and incubated with the appropriate secondary antibody (abcam, Cambridge, UK) for 1 h at RT. They were then washed 3 × 5 minutes in TBS and coverslipped using a mounting medium containing DAPI (abcam, Cambridge, UK). Images were captured at 4x, 10x, and 40x objective lenses on a confocal laser-scanning microscope (Olympus FV1000, Olympus, Japan).

### 2.6. Histology

To examine the intact skin-device interface, devices with attached tissues were embedded in poly(methyl methacrylate) (PMMA). Briefly, the devices with surrounding tissues were fixed in 10% formalin for approximately one week and then dehydrated using an automated tissue processing system (Tissue Tek VIP; Sakura Finetek, Torrence, CA). Next, samples were embedded in PMMA until fully polymerized. The embedded samples were then sectioned (Isomet 4000; Buehler, Lake Bluff, IL), glued to a microscope slide, ground, and then polished (Ecomet 300; Buehler, Lake Bluff, IL). These optically finished slides were then stained with hematoxylin (Sigma-Aldrich, St. Louis, MO) and eosin (Sigma-Aldrich, St. Louis, MO). Briefly, the slides were first stained with hematoxylin for 8 minutes at 60°C and blued under running tap water, and then counterstained with 1% acidified eosin for 2 minutes at 60°C. Stained slides were washed with 70% ethanol, dried, and imaged under a Nikon light microscope (Nikon, Melville, NY).

The length of the down-growing wound margin was calculated by using the following two histological landmarks: (1) 3-phase junction (i.e., the point at which the epidermis, the external environment, and the device surface meet) and (2) position of the skin at surgery (i.e., the start of the porous coating on the post). The length of the exposed coating was measured from the first to the second landmarks and expressed as the length in millimeters. To calculate the GT area, an area measurement tool within the light microscope software NIS Elements D (Nikon, Melville, NY) was utilized. After calibrating the software, 2-D GT boundaries were traced, and the total area was calculated. The downgrowth length and GT areas were obtained in two locations from both sides of the post for a total of 4 measurements per animal and reported as mean ± standard deviation (SD) per group.

### 2.7. Statistical Analysis

All data are reported as means ± SEM unless stated otherwise. As these were clustered datasets with four repeated measurements per animal, a mixed-effects linear regression model was used to account for the lack of independence that could be introduced from data clustering. The significance level was set at *p* < 0.05. All statistical analyses were conducted using STATA (StataCorp, College Station, TX).

## 3. Results

As intended, the characterization data supported relatively pure fluoridated apatites, FA and FHA with some residual carbonates. The ICP-MS results revealed Ca/P ratio of ∼1.62, 1.58, and 1.67, respectively, for HA, FHA, and FA. The FT-IR data showed the presence of carbonate as well as an absence of an OH peak at ∼3500 cm^−1^ for the FA only. The XRD data further confirmed the absence of beta-tricalcium phosphates (*β*-TCP) peaks postsintering at 1150°C.

As stated, a total of 20 female CD® hairless rats were implanted with a single HA-coated device (*n* = 5), FHA-coated device (*n* = 5), FA-coated device (*n* = 5), or noncoated titanium (Ti6Al4V) device (*n* = 5); the latter served as the control. All rats survived without any incidence of infection or inflammation at the implant exit sites during the course of this study (Figure 1). While a small hyper-granulation ring around the post was visible at the implant exit sites in the control group and HA-coated implant groups, FA/FHA-coated groups had no visible signs of hyper-granulation tissues.

**Figure 2:**
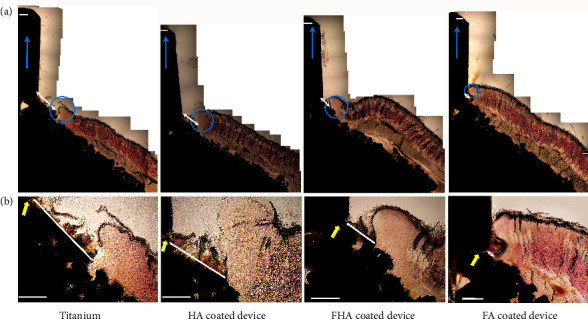
(a) representative set of H&E stained skin-implant interface cross-sections showing degrees of downgrowth (white arrows) and areas of interfacial granulation tissue (blue circles) at four weeks after implantation surgery. (b) Interfaces showing the periprosthetic granulation tissues. Black area represents the implant's cross-section. Scale bar: 500 *μ*m. Implant exit sites point toward the top (Blue arrows). It is also important to note that the area of granulation tissue at the interface decreased with an increasing degree of fluoridation (Ti > HA > FHA > FA). When compared to the controls, the FA-coated surfaces indeed had a significantly lower area of granulation tissues at the interface.

**Figure 3:**
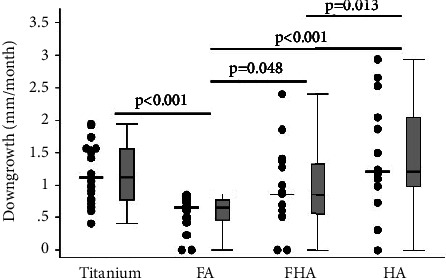
A set of dot- and box-plot showing the degree of downgrowth, which is expressed as mm/month. The data clearly show a statistically significant decrease in downgrowth around FA-coated devices when compared to the uncoated Ti and HA-coated and FHA-coated groups.

**Figure 4:**
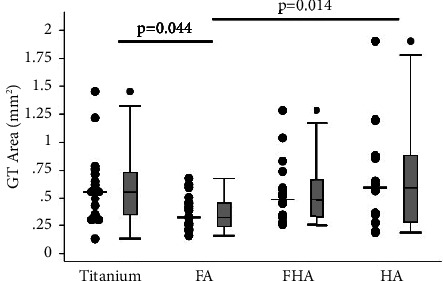
A set of dot- and box-plot showing average granulation tissue regions around the devices at 4 weeks after surgery. The FA-coated devices resulted in significantly reduced GT when compared to the uncoated Ti and HA-coated groups.

This observation was corroborated during the histomorphological evaluations (Figure 2). The data revealed that the FA-coated surface produced the least degree of downgrowth, which was statistically significant (*p* < 0.05) when compared to all other groups (Figure 3). When compared to the control group, the FA-coated implants had significantly reduced granulation tissue regions (Figure 4) and yielded smaller exposed porous-coated regions at the implant base (Figure 2(b)).

**Figure 5:**
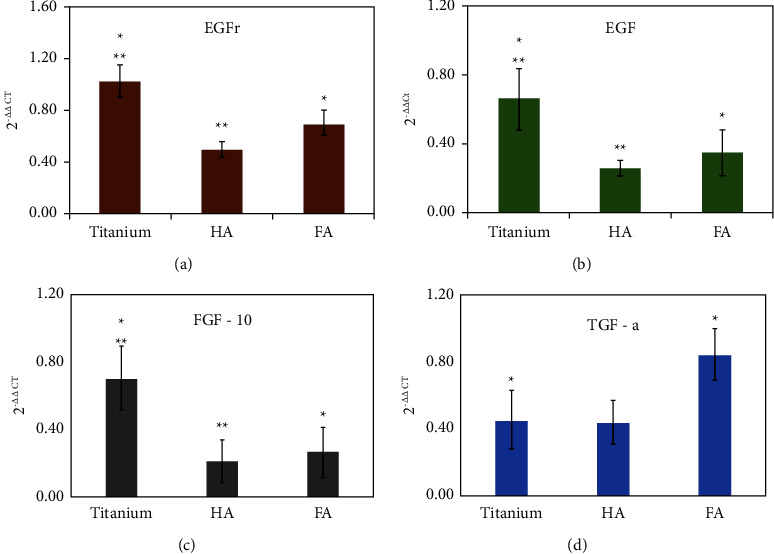
A set of bar charts showing the measured 2 ^ ^ΔΔ^Ct values for EGFr, EGF, FGF-10, and TGF-*α* expression within the granulation tissue (GT) sample from rats implanted with percutaneous devices. Samples were taken from the GT region (Figure 1; yellow circle). EGFr (a), EGF (b), and FGF-10 (c) mRNA were significantly downregulated in the GT around the FA-coated devices, while TGF-*α* (d) was significantly upregulated compared to the uncoated Ti control and HA devices. ^*∗*^ and ^*∗∗*^ indicate statistical significance (*p* < 0.05) between the respective groups.

In order to underpin the mechanisms of healing around the interfaces, as stated within the method, granulation tissue at the interface was isolated, stabilized in RNAlater, and subjected to RNA extraction. After confirming the RNA integral number (i.e., quality), a qPCR was performed. The data indicated that apatite-coated groups (HA and FA) revealed a significant downregulation of EGF, EGFr, and FGF-10 gene expressions when compared to the controls, i.e., uncoated titanium (Figure 5). Interestingly, only the FA-coated group had upregulated expression of TGF-*α* mRNA, indicating its possible role in promoting stomal healing.

**Figure 6:**
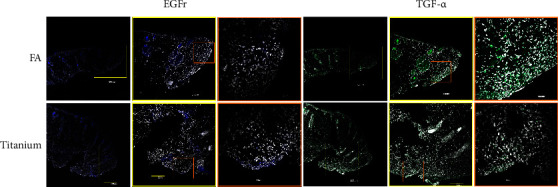
A representative set of EGFr and TGF-*α* confocal micrographs showing the presence of proteins TGF-*α* and EGFr within the granulation tissue. Although proteins are present in the healthy skin appendages such as hair follicles and sweat glands, within the granulation tissue, there were more EGFr and fewer TGF-*α* signals within the controls.

Finally, in order to confirm whether or not mRNA expressions were translated to the protein coding, IF analyses were undertaken (Figure 6). These data corroborate the PCR data. It should be emphasized that both EGFr and TGF-*α* were present in skin appendages within the healthy tissues. However, within the granulation tissue, the trends were as predicted by the PCR data; while FA-coated samples had higher expression of TGF-*α*, the uncoated Ti control samples had higher expression of EGFr.

## 4. Discussion

As stated, this study was designed to investigate the in vivo efficacy of sintered FHA or FA as potential surface coatings for percutaneous devices with the intent to prevent epithelial downgrowth and improve wound healing outcomes, i.e., acceleration of wound healing and wound tissue maturity at the time of sacrifice. The histological data supported the tested hypothesis, in that sintered FA coatings improved wound healing over time as evidenced by relatively lesser amounts of GT tissue indicating greater wound maturity and least epithelial downgrowth at the implant exit site (stoma). Also, qPCR and confocal data further supported the thesis that the FA coating has the potential to improve healing outcomes as evidenced by the downregulation of EGF, EGFr, and FGF-10 while upregulating TGF-*α* within the granulation tissues (GT). These data seem to suggest that such coatings could play a significant role in limiting no-going inflammation at the device interface. Statistical analyses of semiquantitative GT and downgrowth data indicated that they were both significantly (*p* < 0.01) reduced within the FA-coated implant group when compared to the HA-coated and/or Ti (control) implant groups. There was, however, only a slightly significant difference (*p*=0.048) between FA and FHA groups, although the FHA-coated samples had the widest range of values of standard deviation. Thus, FHA-coated implants were not included in further analyses presented in this study.

As previously published, the characterization of the unsintered and sintered powder showed slight calcium deficiencies in HA and FHA powders. However, FA was stoichiometrically pure apatite (Ca:P ratio of 1.67). In FA powders, the absence of the OH peak at ∼3500 cm^−1^ informed that both OH groups of HA were likely replaced by the fluorides [20]. Most importantly, previously published XRD data of the same batch used in this study clearly showed the absence of *β*-TCP within the range of sintering temperatures used in this study [21]. This is an important factor since high temperatures know to induce *β*-TCP phase transitions [22].

With the implantation of any foreign materials, including the implant system used in this study, a foreign body reaction (FBR) is known to occur. Thus, fibrous encapsulation was anticipated around the implants and at the implant exit sites. Although fibrous capsules were seen in and around the deep surfaces of the implant, varying amounts of GTs were present on all surfaces at the implant exit site. This indicated ongoing wound healing, perhaps superseded by the FBR, and may be attributed to the lack of a protective epithelial seal in this area that resulted in the continuing wound healing signals. As unprotected periprosthetic GT is vulnerable to bacteria invasion, over time, infection becomes a more likely outcome. Infection was not evident during this short-duration study. It was also probable that, as GT is a highly vascularized tissue, it can suppress bacterial infiltration and ward-off superficial infection better than relatively avascular fibrous capsules. In addition to mechanistically understanding the microscopic progression of healing GTs, it was deemed important to discern the metabolic processes occurring in soft tissues healing around percutaneous devices possessing differing surface characteristics. To this end, qPCR and IF studies were undertaken.

It is known that GT is an important part of the normal wound healing process, and as the injured tissue continues to heal, it is expected to be remodeled into scar tissue or tissue native to the region; in this current case, the skin. The continuous presence of active GT at the percutaneous postexit site has always been a point of concern. Studies have reported the presence of GT even months after the implantation process regardless of the implant surface type [23, 24]. As stated earlier, it was thought that the inability of stomal epidermal tissue to form a seal with the surface of the protruding implant resulted in the continuous presence of migrating epidermal cells, liming the ability of the periprosthetic tissue to conclude the wound healing cascades, and subsequently, the prolonged presence of GT at the interfaces. The data presented in this study clearly showed that while the apatite coating does improve and resolve healing within the GT tissue region, the Ti group had the largest GT region within all tested groups (Figures 2 and 4). This finding supports the postulation that cell-to-implant integration could resolve the healing cascades at the 3-phase junction defined as where the soft tissue, implant material, and external atmosphere meet.

The connection of the tooth with the surrounding gum tissue may be an analogous circumstance and relevant to the topic of percutaneous material research since the tooth has one of the few natural surfaces that has a functional connection between a nonliving but biological surface and living tissue. Thus, apatite material, in particular HA, has been used as a coating material in the clinical application of various percutaneous devices. These include the ITAP [25, 26], dental implants [27] and bone-anchored hearing aids (BAHA) [28]. Unfortunately these implants all show limited clinical success. The plasma coating process was generally used to produce these devices yields, around 60–70% crystallinity [29]. It is believed that healing cascades within GT may facilitate the resorption of such HA coating and perhaps the reason for its' limited success [30]. It is, however, interesting to note that FHA and FA have been reported to have slower bio-resorbable properties than that of HA [9].

While ongoing wound healing ultimately ends in the protection of deep tissues, in the presence of a percutaneous post, the result of a failure of soft tissue/surface integration ultimately yielded epithelial down growths [1]. As anticipated, with Ti surfaces, there was no integration of epithelial cells with the interface, and thus, the downgrowth was profound and occurs at a faster rate. As the dental literature points out, enamel surfaces (i.e., FHA in humans and FA in sharks) can induce cellular changes to the adjacent junctional epidermal cells to code for the production of hemidesmosomes [13]. Moreover, other researchers postulated that the other junctional proteins, such as, Laminin 332, and integrin *α*6*β*4 can also form ligation against the nonliving calcium-rich enamel surface [31–33]. Thus, we expected apatite-coated devices to mimic enamel surfaces. As predicted, downgrowth was limited in FHA and FA groups (Figure 3). Although HA might be expected to produce a similar outcome, limited downgrowth was not seen. As pointed out earlier, as HA is bio-resorbable, with the progression of resorption, the underlying Ti surface could gradually have become exposed [23, 34, 35].

Previous studies have shown that FGF-10, EGF, EGFr, and TGF-*α* are all differentially expressed within the GT when compared to the healthy skin (i.e., uninjured/unmodeled dermal tissue) [36–40]. To better understand the progression of healing around the implants, mRNAs were quantified using qPCR. This PCR data clearly showed reduced expression of FGF-10 and EGF within the GTs of the FA group (Figure 5). FGF-10 is a well-known multifunctional mesenchymal-epithelial signaling growth factor and is implicated in activities associated with several intracellular signaling cascades [41], resulting in cell proliferation, differentiation, and invasion [42]. During wound-healing, FGF-10 is considered essential for epithelial development and promotes keratinocyte motility, survival, and activation [43, 44]. Although not conclusive from our data, the reduced presence of FGF-10 perhaps indicates the direction of keratinocyte differentiation and maturation [45] rather than continuously being present as a migratory phenotype as previously reported [23, 46]. Interestingly, one mouse study reported overexpression of FGF-10 one day after the injury, which decreased rapidly by three days, illustrating its importance in early wound healing [47, 48]. FGF-10 is also implicated in corneal endothelial wound healing [49]. These studies collectively indicate that FGF-10 is necessary for epithelial migration and promotes healing. Our data showed more than 50% reduction in the expression of FGF-10 in the FA group when compared to the Ti group (control). The observed downgrowth differences appeared to suggest that periprosthetic tissues in the FA group might have progressed further toward the completion of wound healing. Moreover, judging the periprosthetic tissue responses, as indicated by the histological organization of vascularity within the GT, the FA group indeed appeared to have a more structured 3-phase junction, supporting the PCR (FGF-10) data.

It is well known that EGFr and EGF play an essential role in wound healing [50]. Again, EGF is widely expressed during early wound healing phases, and it is also essential for epithelial development and migration [51]. The EGF also has multiple functions, including fibroblast and endothelial cell stimulation, proliferation, and migration [40, 50]. It binds to the EGFr, which is expressed in the majority of cells in the skin. When bound, EGFr with EGF together activates phosphorylation. The reduced presence of EGFr and EGF in the apatite-coated group (Figures 5 and 6) may suggest that cells within the granulation tissues are less migratory (progression towards the conclusion of wound healing), and this reduced presence perhaps plays a vital role in limiting the downgrowth. This observation needs further validation in a time-course model.

Another member of the EGF family is TGF-*α*, which is known to play an important role in the wound repair process. It is only upregulated in the FA group compared to the other tested surfaces [52]. It is known that TGF-*α* is a mitogenic polypeptide that can bind to EGFr to promote anchorage-independent cell proliferation [53]. In normal wound healing, TGF-*α* is expressed during the re-epithelialization phase. In combination, EGF and TGF-*α* are supposed to increase keratin 6 and 16 coding within the keratinocytes, improving healing [54]. The presence of increased levels within the relatively healed periprosthetic tissues of the FA group is a little puzzling. Although overexpression of TGF-*α* is implicated in several signaling pathways, in this study, it may indicate the end of the cell migration phase, leading to a healed stoma. One study showed that eosinophils, present in the healing wound, produce TGF-*α*, at seven days after injury [55], suggesting that periprosthetic tissues around the FA-coated implants had moved toward completion of the healing phase. Further studies are needed to confirm its role in stomal healing. Perhaps, a total RNA sequencing study would yield a better understanding of periprosthetic tissue healing.

### 4.1. Limitations

This study focused on the use of FHA and FA as coatings for improved healing around percutaneous devices with the reduction of downgrowth and reduction in granulation tissue volumes at the interface as the outcome measures. There are a few limitations to this study. This animal study was only used as a proof of concept. It is important to note that the healing cascades in rats are different than those in humans [56–58]; thus, a more relevant translational model, i.e., a swine model with time-course study, might be used to understand their translation to human applications. Although the implant was percutaneous, it was not anchored to bone. Percutaneous tissues that are anchored to the underlying bone can endure relatively higher mechanical shear forces at the skin/implant interface. Thus, in order to understand the effects of anchoring percutaneous devices to bone (i.e., osseointegration) as well as any shear forces related to ambulation, a weight-bearing model is warranted. We are planning to test this coating in our established pig and sheep model [23, 46]. The mechanical and chemical surface stability of FA coatings on titanium devices, over time, is another concerning variable that remains uninvestigated. Thus, a degradation study of the coating should be carried out to understand the long-term durability of this material as a coating. It is also worth noting that, as 18%–25% infection rates are associated with the human percutaneous devices [59, 60], a more reverent infection animal model is also needed. This study does not address coating ability to protect the periprosthetic tissue from the potential of natural or experimentally-induced infection associated with percutaneous implants. The performance of coating in the infection model therefore needs to be addressed in our future studies. Finally, the reason for the limited down growth seen in the FA group was possibly due to the surface-mediated induction of hemidesmosomes, which was not determined in this study. This was due to our inability to obtain thinner sections of the intact implant-soft tissue interface for TEM analysis. Future studies are needed to confirm the presence of hemidesmosomes at the 3-phase junction. Regardless, the FA-coated devices appeared to establish a stable stoma and limit the downward migration of the epidermis.

## 5. Conclusion

Percutaneous devices coated with FA did show improved wound healing and downgrowth outcomes compared to the uncoated titanium and HA-coated devices. The PCR data showed differences in the expression of the healing cascades between percutaneous uncoated titanium devices and those coated with FA, suggesting a stable stoma.

## Data Availability

The data that support the findings of this study are available in the VA intranet system; however, these data can be made available to any individual upon request to the corresponding author.
